# Storytelling for impact: the creation of a storytelling program for patient partners in research

**DOI:** 10.1186/s40900-023-00471-0

**Published:** 2023-07-25

**Authors:** Leah E. Getchell, Marian Reich, Selina Allu, Cathy Woods, Teresa Atkinson, Mary Beaucage, Leanne Stalker, Dwight Sparkes, Catherine Turner, Audrey L’Esperance, Kevin Burns, Meghan J. Elliott, Helen Chiu, Norman D. Rosenblum, Ruth Sapir-Pichhadze

**Affiliations:** 1 Can-SOLVE CKD Network, Vancouver, Canada; 2 Canadian Donation and Transplantation Research Program, Edmonton, AB Canada; 3 Kidney Foundation of Canada, Montreal, Canada; 4 Health and Social Services Management, École nationale d’administration publique (ENAP), Quebec City, Canada; 5 Division of Nephrology, Department of Medicine, The Ottawa Hospital, University of Ottawa, Ottawa, Canada; 6 Department of Medicine, University of Calgary, Calgary, Canada; 7 BC Renal, Vancouver, Canada; 8 Division of Nephrology, The Hospital for Sick Children, Department of Paediatrics, University of Toronto, Toronto, Canada; 9 Centre for Outcomes Research and Evaluation, Research Institute of McGill University Health Centre, Montreal, QC Canada; 10 Department of Epidemiology, Biostatistics, Occupational Health, McGill University, Montreal, QC Canada; 11 Division of Nephrology, Department of Medicine, McGill University, Montreal, QC Canada

**Keywords:** Patient partnerships, Patient and public involvement, Patient engagement, Storytelling, Health research, Patient-oriented research, Kidney, Kidney transplant, Organ donation, Living organ donation

## Abstract

Storytelling is a powerful means to evoke empathy and understanding among people. When patient partners, which include patients, family members, caregivers and organ donors, share their stories with health professionals, this can prompt listeners to reflect on their practice and consider new ways of driving change in the healthcare system. However, a growing number of patient partners are asked to ‘share their story’ within health care and research settings without adequate support to do so. This may ultimately widen, rather than close, the gap between healthcare practitioners and people affected by chronic disease in this new era of patient and public involvement in research. To better support patient partners with storytelling in the context of a patient-oriented research network, Canadians Seeking Solutions and Innovations to Overcome Chronic Kidney Disease (Can-SOLVE CKD) Network adapted an existing in-person storytelling workshop for patient educators within a hospital setting. The result is a 6-week virtual program called *Storytelling for Impact*, which guides patients, family members, caregivers and organ donors in developing impactful stories and sharing them at health care and research events, e.g., conferences. The online series of synchronous workshops is co-facilitated by story coaches, who are program alumni and Can-SOLVE CKD staff with trained storytelling experience. Each story follows a structure that includes a call to action, which aims to positively impact the priority-setting and delivery of care and research in Canada. The program has been a transformational process for many who have completed it, and numerous other health organizations have expressed interest in sharing this tool with their own patient partners. As result, we have also created an asynchronous online program that can be used by other interested parties outside our network. Patient partners who share their stories can be powerful mediators for inspiring changes in the health care and research landscape, with adequate structured support. We describe two novel programs to support patient partners in impactful storytelling, which are applicable across all health research disciplines. Additional resources are required for sustainability and scale up of training, by having alumni train future storytellers.

**Supplementary Information:**

The online version contains supplementary material available at 10.1186/s40900-023-00471-0.

## Abstract (plain language summary)

Storytelling is a powerful means to evoke empathy and understanding among people. When patient partners share their stories with health professionals, this can prompt listeners to reflect on their practice and consider new ways of improving the healthcare system. However, as a growing number of patient partners are asked to ‘share their story’ within health care and research settings, there is often not enough tools and resources to support them in preparing their stories in a way that will be impactful for the audience members. Our kidney research network sought to create a novel in-person storytelling program to address this gap within our health research context. The result is a 6-week program called Storytelling for Impact, which guides patient partners—which includes patients, family members, caregivers and organ donors—in developing impactful stories and sharing them in a formal setting. The program is led by story coaches, who are patient partners and staff with trained storytelling experience. Participants are encouraged to include a call to action in their story, which aims to outline clear ways in which health professionals can facilitate positive change in health research or care. Many participants have described the program as transformational, and numerous other health organizations have expressed interest in sharing this tool with their own patient partners. As a result, we have also created a second online program that can be used by other interested parties outside our network. This paper highlights the adaptation process, content, participant feedback and next steps for the program.

**Supplementary Information:**

The online version contains supplementary material available at 10.1186/s40900-023-00471-0.

## Background

Over recent years, there is increasing demand to transform the healthcare system by incorporating patient-identified priorities into the research enterprise in the context of patient-oriented research. Given this new focus, there has been an increase in demand for patients, family members, caregivers and organ donors (herein referred to as patient partners) to ‘tell their story’ in various settings, including at research team meetings, training events and conferences. While formal and informal training programs and resources have been developed to educate patient partners on the research process and how to work with interdisciplinary teams [1], none of these programs specifically prepare patient partners to share their healthcare stories in a meaningful and impactful way.

Without supporting patient partners in the preparation of sharing their lived experience within the health research setting, stories may run the risk of being unfocused or poorly received because the intention of the story may not be clear upfront [2, 3]. Failing to provide adequate support in storytelling could have potentially negative effects in terms of building meaningful relationships, which are critical to successful patient-oriented research [4]. In this paper, we describe the development and implementation of a unique storytelling training program for patient partners, as well as highlight the impact this training has had on those who have engaged with the training, and the lessons learned.

## Theoretical context

The design of the program and curriculum we outline herein resonates with concepts from *narrative medicine* [5] to support the creation of more empathetic relationships between clinicians and patients. Medicine practiced with narrative competence recognizes, absorbs, interprets, and is moved by the stories of illness. [6]. This unique clinical method can be used to teach practitioners to deeply understand the experience of illness and care through the telling and receiving of patient stories. Notably, it provides a key opportunity to shed light on matters that strongly affect patients, but that providers may not think about on a daily basis, such as challenges related to inequity in health or transportation to dialysis clinics. Some key reasons for sharing stories include: to listen, honour and learn from those who have experienced illness and care; to inspire ideas for positive change in the health care system; and to promote healing relationships and humanistic, patient and family-centered care. These stories, usually shared in-person in a clinical setting, help to remind medical practitioners of the foundational values of patient-centered care, defined by the following principles and behaviours: dignity and respect, communication and information sharing; collaboration and empowerment and; comprehensive and coordinated care [3]. These narrative medicine techniques infused with the patient- and family-centered care values can be a key factor in helping advance a better understanding of the patient experience, improving quality and safety, and shifting cultural norms in health care and health research environments [3].

Within the context of our national kidney research network, hearing patient stories during the research stages can be particularly helpful in ensuring that research priorities align with the interests of patients, families, caretakers and organ donors.

## Storytelling: a priority among network members

Can-SOLVE CKD Network, a national patient-oriented kidney research network in Canada, enables patient partners to work alongside researchers and policy makers to advance kidney health research. In the spring of 2017, following one of the first network meetings where patients were in attendance, there was a sense of discord after meeting. Neither the audience nor patient partners were adequately prepared for the sharing of stories. In post-meeting conversations, patients expressed a perceived lack of understanding from researchers. Researchers, on the other hand, expressed difficulty coping with the strong emotions that patients expressed while sharing their stories. It became clear that if story telling were to happen again without at least better support for the patients, efforts to build bridges in these early years of patient-oriented research could be significantly jeopardized. In a resulting network-wide training needs survey in the summer of 2017, ‘telling impactful stories’ was ranked second out of five identified priorities among network members. This prompted the Can-SOLVE CKD’s Training and Mentorship Committee (TMC) [7], a committee composed of patient partners, researchers, clinicians, and community members tasked with developing capacity-building tools, to explore the idea of creating a storytelling tool.

Can-SOLVE CKD worked collaboratively with the Patient Experience Office at the London Health Sciences Centre (LHSC) that had been delivering a storytelling workshop to prepare patients to share their stories with professional healthcare audiences. A staff member from LHSC’s Patient Experience Office, and a patient partner who had completed the in-person storytelling workshop, were subsequently invited to the Can-SOLVE CKD’s annual in-person TMC meeting in January of 2018. The LHSC representatives outlined the narrative medicine and patient-centered care foundations of their workshop format and shared the impact the curriculum had on both audiences and storytellers [8]. Members of the TMC agreed to collaborate with consultants to help the network adapt their storytelling curriculum to fit our national patient-oriented research context.

**Fig. 1:**
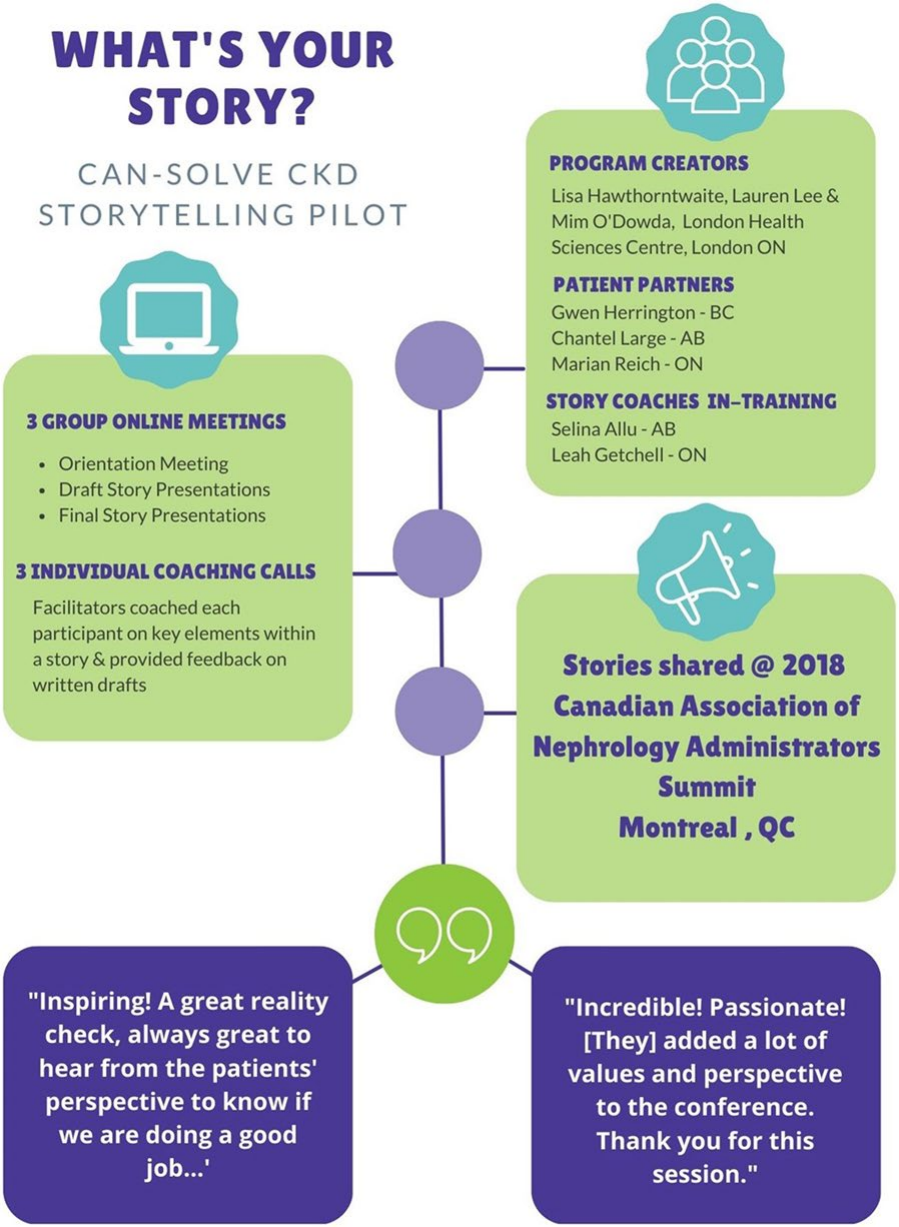
Illustration of the Storytelling for Impact pilot program in 2018

The adapted version by Can-SOLVE CKD was designed to take place online over a 6-week period, and include three online Zoom meetings, along with individual coaching calls. Between February and April of 2018, the TMC worked with the LHSC consultants to adapt the delivery format of the original content and roll out a pilot version with two staff members trained as coaches and three network patient partners who were already slated to share their story at the Canadian Association of Nephrology Administrators annual meeting in May of 2018 (Fig. 1).

It was during the delivery of our pilot that we recognized the importance of including a call to action—hence the renaming of the Can-SOLVE CKD version to *Storytelling for Impact*. Including a call to action (Table 1) as part of the patient story was meant to spark imagination, innovation, and new direction in the hearts and minds of the audience, who are typically health researchers, clinicians and policy makers. This explicit call to action was a new addition to the program and a great example of how we adapted the content to also fit our patient-oriented research context. When we allow patients’ stories to be heard, we delve into expertise the clinicians and practitioners rarely possess—lived experience of the illness they are studying. This brings fresh perspective to the research table with vision to enhance the work.

**Table 1:** Examples of calls to action from past sessions of *Storytelling for Impact*

Example #1	Better quality of life for dialysis patients
Example #2	Increasing kidney donor health awareness
Example #3	Mental health aspect of chronic illness is overlooked because the acute physical symptoms often take priority
Example #4	Remembering the power and simplicity of human touch are more important to me as a patient then the latest technology or the best medical care provided. Humanity, dignity, respect and the greatest of these is humanity
Example #5	What can be done to improve how indigenous patients are treated in the health care system in Canada?

In Can-SOLVE CKD’s adapted *Storytelling for Impact* program, participants are asked to focus on moments of care within their healthcare journey. Participants identify which parts of their story fit under the foundations of patient-centered care [3]. The goal in this approach is to not only help identify which patient- and family-centered values and behaviours are present or lacking, but provide the opportunity to reflect and uncover a deeper meaning and understanding of their experience, rather than simply retellingevents in a chronological manner. In pinpointing the underlying theme or identifying a pivotal moment within their healthcare journey, the storytellers are then asked to create a call to action for their audience. To assist participants in this process, we created a Storytellers Handbook, which includes a template to help them capture the key messages in a single page, starting with having them reflect on their call to action and finding moments of care along the way that illustrate the need for their call to action (Additional file 1: Appendix 1). Notably, a key goal of patient-oriented research was to influence change in the healthcare system; therefore patient partners providing a call to action while sharing their stories was a clear way to link the storytelling back to the foundational values of our patient-oriented research network.

## Storytelling for impact: overview

Once participants express interest in participating, they are contacted by Story Coaches to have a one-on-one phone call where the goals of the program are discussed along with the timeline (Table 2). They can ask any questions about the upcoming sessions and are given the Storytellers Handbook, which includes resources and worksheets to help them in preparing their story.

**Table 2:** Overview and timeline of *Storytelling for Impact* program

Week 1	Orientation: meet the group, hear alumni stories, walk through independent work required, questions and discussion
Week 2 & 3	Write & submit first draft of story
Week 4 & 5	Story coaches review & have one-on-one calls with participants
Week 5	Write second draft of story: focusing in on clarity of two moments of care and their ‘call to action’. Storytellers begin practicing at home
Week 6	Story Circle. All participants reconvene with Story Coaches and Alumni. Storytellers present their stories and receive peer feedback
Week 7 & 8	Final phone call with Story Coaches for additional support and feedback in preparation for public presentation

Next, all participants gather for the first online zoom meeting led by the Story Coaches. At this 2-h session, participants hear stories prepared by workshop Alumni, background information on the importance of storytelling across cultures, an introduction to moments of care within the patient- and family-centered care framework [9], as well as the importance of considering the call to action within the context of their audience.

During this first group call, participants are warned about the potential emotional toll of returning to what may be very difficult events in their lives and are invited to reach out to Story Coaches if they are feeling overwhelmed and would like to be directed to appropriate community supports. Participants are also encouraged to take time to process and honour the emotion when it does come [3].

Resources from the Storytellers Handbook are reviewed, and participants leave with the tools and information they need to write the first draft of their story. They are then given 2 weeks to submit the first drafts of their stories, at which point Story Coaches review for theme clarity, moments of care and call to action, and provide their feedback to participants. Stories are expected to range from 750 to 1000 words, which typically translates into a 5-to-6-min oral story.

Following the first round of revisions, participants work over the following 2 weeks to refine their story and begin practicing their presentation at home in preparation for the Story Circle. In this final virtual session, all participants reunite, as well as the Story Alumni from the first session, to hear each others’ stories. Peers provide feedback in real-time, highlighting elements of the stories that impacted them, and providing suggestions for improvement (presentation skills, tone, language, speech rate, etc.). Feedback is also recorded on a unique template, which is collated and sent to the participant by the Story Coaches after this session. A final one-on-one session between each participant and the coaches allows the new storyteller to discuss their experience, as well as present their story again after incorporating the feedback from their peers. Final discussion is had on any outstanding preparations that might be needed ahead of a specific public speaking event and storytellers are given the Facilitators Handbook, a small document meant to support the event organizers in creating the best environment possible for the patient’s story to be heard within the context of their in-person or virtual meeting or conference. Lastly, a final evaluation link is sent to gather formal data on the experience of the storyteller and to support future iterations of the *Storytelling for Impact* program. Officially a Story Alumni, the participant is added to a growing community of patient partners who have completed the program and who have the skills and tools to craft and share impactful stories geared to a particular audience, e.g. other patient partners, researchers, clinicians, practitioners and policy makers. These individuals are now eligible to become Story Coaches in the program if they so wish.

## Strengths

There are several strengths in adapting and re-designing the workshop. Critically, as a patient-oriented research network, Can-SOLVE CKD has many patient partners who help design and implement training materials within the network, and this is true for the *Storytelling for Impact* program as well. Their first-hand experience is essential for ensuring that educational materials and resources are appropriate, relevant, and accessible for other patients. The greatest strength for alumni who have completed the program is that they have a refined, polished story ready for presentation, and one that is impactful with a call to action for healthcare professionals. When patient partners tell impactful stories with clear objectives at research-related events, e.g., conferences, this can help ensure their lived experience informs research priorities and is translated into pragmatic research questions. The feedback from participants has been overwhelmingly positive, and suggests that the program meets its overarching goals (Logic Model, Additional file 2: Appendix 2). This is illustrated in several testimonials from participants.

### Testimonial #1 (Mary Beaucage, patient partner)

*“Storytelling For Impact* challenged me to look at a few events in my life and how I could bring them together and articulate a call to action, or really make my audience feel the importance of what I wanted them to remember. For me, that meant I had to dig deep and examine the systemic racism I experienced in my care. I shared how it made me feel, but also I wanted to challenge the audience to examine their own biases…. I think it really helped put a face on systemic racism for them and my story changed the people who heard it that day… Storytelling is part of our tradition as Anishinaabe people. Sharing the things I’ve gone through and being vulnerable helps build relationships, whether it’s with other patients, researchers or policymakers. I’m hoping that every time I share my story it will help bring change and move the needle for those with chronic kidney disease and who need a transplant.”

### Testimonial #2 (Teresa Atkinson, patient partner)

As a long-term patient, I had shared my story in health care settings several times before and I didn’t think I really needed a course to help me tell my story. Then I was challenged to condense my 38-year story of living with kidney disease into a 7 min presentation. This seemed like an insurmountable task until I was introduced to *Storytelling for Impact*. This course challenged me to think about my story in different ways. What was truly important to share in that 7 min? What would have the most positive impact on the audience? What did I really want them to take away and do after I shared my story? After taking this course I feel more empowered to share my story and make an impact on the healthcare system. Storytelling for Impact is an excellent program I regularly recommend for other patient partners to consider.

### Testimonial #3 (Cathy Woods, patient partner)

“The storytelling program helped me find my voice, assisting me to tell my kidney story succinctly and know how to reach my audience and introduce them to my kidney journey. It gave me the confidence to speak in front of hundreds of people with the support of all my Can-SOLVE CKD colleagues. I found my place and know why sharing my story is so important to reach out to others with similar experiences and impact kidney research to improve outcomes for all… I recall many health care folks, including one doctor say to me after a meeting, ‘Cathy we learn so much from you every time you speak.’ That meant the most to me, they truly listened to changes I was suggesting and validated my reason for being a patient partner and my role within Can-SOLVE to make changes that will benefit all families that experience and live with kidney disease.”

### Testimonial #4 (Arlene Desjarlais, patient partner)

Taking the Storytelling program has been completely life changing for me… I am forever humbled and it has made losing the love of my life a little bit easier as it gave me the chance to share our story and the knowledge that I know there is another family out there who will hear our story and know they are not alone during their journey. My Glen will never be a statistic and will live as large in death as he did in life.”

## Asynchronous program

The initial *Storytelling for Impact* program developed by Can-SOLVE CKD is human resource intensive and can only appropriately support up to six patient partners in a cohort. Therefore, we have developed an asynchronous online module with the content of the Storytellers handbook that can be accessed by patient partners both within our network and beyond, on their own time. This was officially launched in November 2022.

## Limitations

While Can-SOLVE CKD staff initially led the training sessions, patient partners who had completed the *Storytelling for Impact* program were then recruited and trained as Story Coaches to co-facilitate subsequent sessions. The motivation behind this format is two-fold. Firstly, the patient partners involved in the early iterations of this program felt it was a critical element to have at least one Story Coach who has gone through the storytelling process themselves, enabling peer-to-peer feedback. Secondly, this format supports the spread and scale of the tool. Thus, a current limitation of program is the absence of a formal structure to onboard and support Story Alumni in becoming Story Coaches. While a Story Coaches handbook has been created to support new coaches to some extent, we have been focused on the implementation of the asynchronous format. Additional resources, including time and personnel are needed to onboard and support Story Alumni to transform into Story Coaches.

As well, it is important to note that while steps are taken to address the emotional toll of revisiting stories during the synchronous workshop, one of the limitations of the asynchronous model is not having the ability to monitor and/or support participants emotionally.

Another limitation of our online delivery format, and a divergence from the original in-person format, is our inability to be directly involved as facilitators of these polished stories when they are presented. Hawthornthwaite et al. [3] speak about the ‘three sides to every story’ and the important relationship between the patient storyteller, the facilitator of the event, whose job it is to create a safe container for the story, and members of the audience themselves, who must engage in critical reflection and dialogue after the story is shared, as this is where the real learning can take place. Since our version has been designed to support our network of national patient partners, it is impossible for us to always participate in the wide variety of in-person and virtual events our Story Alumni have been invited to. To address this limitation, at the suggestion of one of our Story Alumni, we have also created a Facilitators Guide, which can be shared with event organizers. The document includes an overview of the facilitator’s role in creating appropriate context and a safe space for the patient storyteller, as well as preparatory checklists, which include discussing the goal of having the patient story included in the session, the overall goal of the event/meeting and who will be in the audience. While our current workshop focuses on preparing the patient storytellers, our hope is that this additional guide may help fill the gap in preparing the story facilitator and thus the audience to receive and learn from the patient story.

With the “three sides to every story” concept in mind, it is also important that researchers are open and willing to hear patients’ stories and learn from them. While our workshops focus on training for patient partners, it’s important to emphasize the need for more training and awareness among researchers on the value in listening and learning from patient partners, which is beyond the scope of this paper.

As well, Inclusion, Diversity, Equity and Accessibility (IDEA) is critical in health research, and an important cornerstone of Can-SOLVE CKD’s work. Therefore we have a dedicated council of Indigenous people and an IDEA working group, who provide guidance and participate in our programs. The patient partners of Can-SOLVE CKD therefore reflect people of diverse backgrounds, with equally diverse stories to share with healthcare professionals. Recruitment of diverse peoples—and especially underrepresented groups—may be challenging for other organizations without this structure, but is still very important in ensuring that underrepresented peoples’ stories are told. We recommend that people who offer storytelling workshops take extra care to recruit participants from underrepresented groups.

Lastly, patient-oriented research is a multi-faceted process whereby patients and researchers work together to find healthcare solutions. While patient storytelling is one method to support meaningful engagement and partnerships, additional training, support, and culture change are needed to facilitate true, patient-engaged research in the kidney space.

## Future directions

Currently, Story Coaches are not yet available to support the participants in the asynchronous online module; however, we will be training and supporting our Story Coaches to offer virtual support of the asynchronous participants in the coming year. This will be an important means for the Story Coaches to then co-lead future iterations of the synchronous program. With this, we hope to expand into a train-the-trainer model so those beyond the network will be able to participant in the synchronous program, and subsequently take it back to host in their own community.

Although the workshops have only been recently launched, we plan to collect some data moving forward, for example capturing the number of storytelling modules offered, participants, facilitators trained, and presentations by storytellers.

## Conclusion

Patient storytelling is a valuable means for advancing patient-centred care, and it is necessary to provide patient partners with adequate guidance and coaching on how to effectively share their stories with health professionals. Can-SOLVE CKD Network adapted a structured program to support patient partners in storytelling, that provides patients, family members, caregivers, and living organ donors with a structured means for sharing their healthcare experiences. The program, available in a synchronous or asynchronous format, could be used more broadly in the future by patients outside of the Can-SOLVE CKD network to advance patient voices more effectively in health research and care.

## Supplementary Information

**Additional file 1.** Storytelling for Impact.

**Additional file 2.** Appendix 2 – Logic Model.

## Data Availability

Not appliacble.

## Abbreviations

Can-SOLVE CKD: Canadians Seeking Solutions and Innovations to Overcome Chronic Kidney Disease
LHSC: London Health Sciences Centre
TMC: Training and Mentorship Committee
