# Supplementary material for: Storytelling for impact: the creation of a storytelling program for patient partners in research
Source: Res Involv Engagem. 2023 Jul 25;9:57. doi: 10.1186/s40900-023-00471-0 (PMC10369735; doi:10.1186/s40900-023-00471-0)
Supplement: Supplementary file 1 — Additional file 1. Storytelling for Impact. [file 40900_2023_471_MOESM1_ESM.docx]

**Storytelling for Impact**

**Planning Template**

| **Begin with the ending**  What do you want your audience to know and do at the end of your story |
| --- |
| Choose a moment of care which leads them there… |
| Choose a second moment which supports your call to action! |
| **Introduction**  How will you set up your story? How much of your healthcare journey does the audience need to understand? Which details will best support your call to action? |
