# Supplementary figures and images for: Storytelling for impact: the creation of a storytelling program for patient partners in research
Source: Res Involv Engagem. 2023 Jul 25;9:57. doi: 10.1186/s40900-023-00471-0 (PMC10369735; doi:10.1186/s40900-023-00471-0)

Appendix 2 – Logic Model


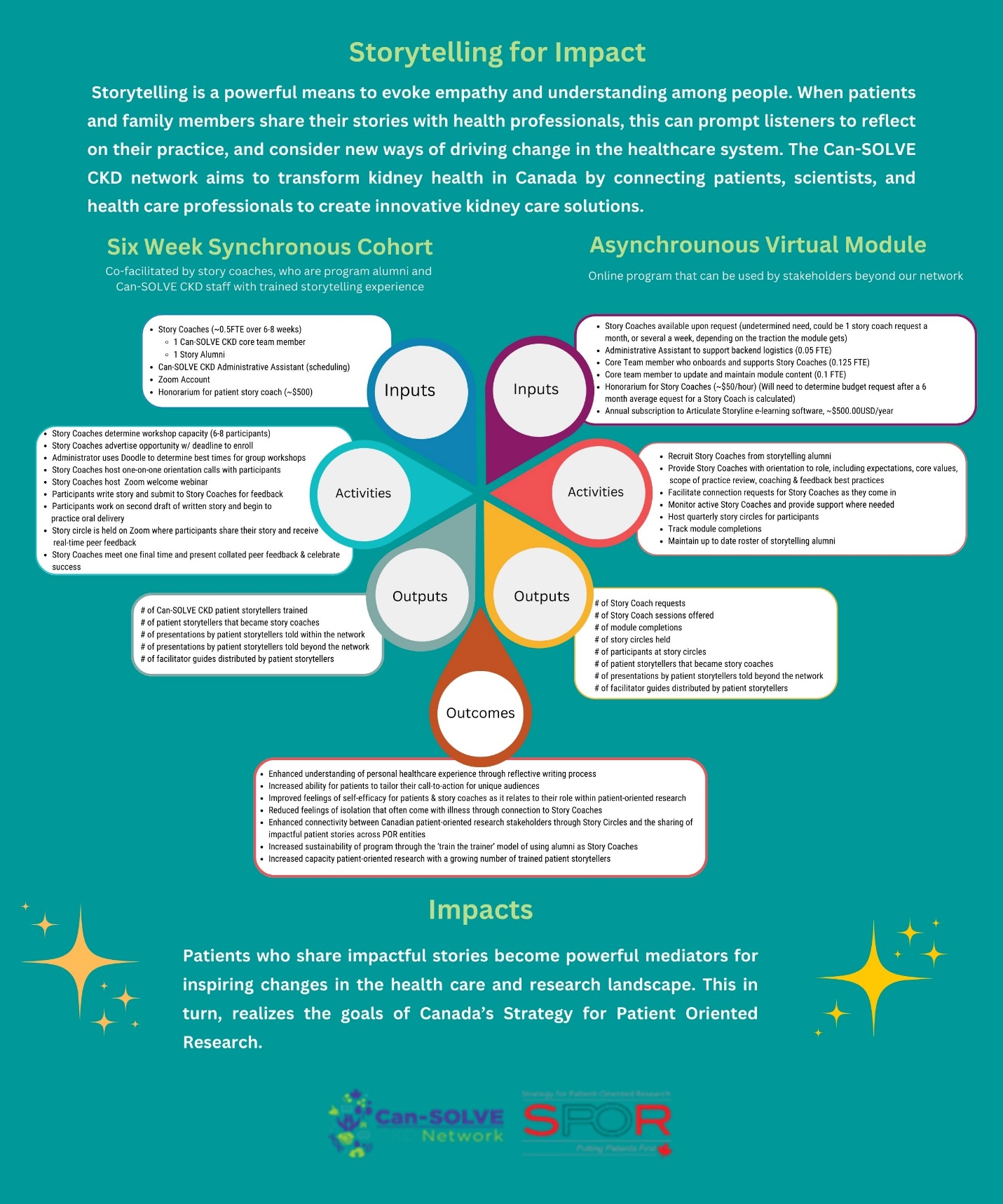

Supplement: Supplementary file 2 — Additional file 2. Appendix 2 – Logic Model. [file 40900_2023_471_MOESM2_ESM.docx]
